# Effects of the amino acid mixture on growth performance, non-specific immunity and intestinal health in Pacific white shrimp (*Litopenaeus vannamei*)

**DOI:** 10.1016/j.cirep.2026.200282

**Published:** 2026-04-19

**Authors:** Chengjie Lv, Jiali Wu, Yongliang Liu, Weiwei Zhang, Dinglong Yang, Jianmin Zhao

**Affiliations:** a Key Laboratory of Coastal Biology and Biological Resources Utilization, Yantai Institute of Coastal Zone Research, Chinese Academy of Sciences, Yantai, 264003, PR China; b Muping Coastal Environmental Research Station, Yantai Institute of Coastal Zone Research, Chinese Academy of Sciences, Yantai, 264117, PR China; c School of Marine Sciences, Ningbo University, Ningbo, 315832, PR China

**Keywords:** *Litopenaeus vannamei*, Amino acid mixture, Growth performance, Immunity, Intestinal flora

## Abstract

In the present study, a mixture of amino acids (NutriMix, composed of 42% arginine, 39% lysine, and 19% methionine) was used to evaluate its effects on growth performance, non-specific immunity and intestinal health in Pacific white shrimp (*Litopenaeus vannamei*). In a 42-day feeding trial, shrimp with an initial weight of 2.45 ± 0.03 g were divided into six groups: a basal diet control (CT), three graded levels of amino acid mixture (0.5‰, AL; 1‰, AM; 2‰, AH) and two low-fishmeal diets supplemented with 1‰ (FM) and 2‰ (FH) amino acids. The results showed that the weight gain rate and specific growth rate were significantly increased in the AH group (*P* < 0.05). Meanwhile, the immune defense ability of hemocytes was effectively enhanced by NutriMix, as evidenced by maintained ROS homeostasis, elevated phagocytic activity, and reduced apoptosis rate (*P* < 0.05). Moreover, NutriMix upregulated the expression of antioxidant genes (*GPX*) in gills, and increased the activities of POD, ACP, and AKP in hepatopancreas, thereby improving the antioxidant capacity and immune function of tissues. For intestinal health, NutriMix enhanced intestinal antioxidant enzyme activities, optimized nutrient absorption and strengthened the intestinal barrier, especially in the AH group. 16S rRNA sequencing showed that NutriMix improved gut microbiota structure, elevated the relative abundance of Bacteroidota, inhibited *Vibrio* proliferation and reduced inflammatory risk, meanwhile, *Lysobacter* and *Xanthomarina* showed significantly positive correlations with the growth of shrimp. In conclusion, 2‰ dietary amino acid mixture improves growth, systemic immunity and intestinal health of shrimp, even under low fishmeal conditions. This study provides a theoretical basis for applying amino acid mixtures in shrimp aquaculture, and future research can focus on their molecular mechanisms and practical application effects.

## Highlight

• NutriMix, a blend of 42% arginine, 39% lysine and 19% methionine, significantly boosts growth in Pacific white shrimp (*Litopenaeus vannamei*).
• Dietary supplementation with 2‰ NutriMix can enhance immune response and antioxidant enzyme activities in *Litopenaeus vannamei*.
• NutriMix improves intestinal health and regulates gut microbiota in Pacific white shrimp (*Litopenaeus vannamei*).

## Introduction

The Pacific white shrimp (*Litopenaeus vannamei*) is a high economic value aquaculture species, known for its fast growth rate, strong adaptability to a wide range of salinities and temperatures, excellent disease resistance, tasty meat and high nutritional [1]. These conspicuous characteristics have made it one of the most popular shrimp species cultured worldwide, especially in China, where it has become a significant contributor to the aquaculture industry [2]. Fishmeal (FM) is a high-quality protein source for commercial *L. vannamei* feed, owing to its abundant amino acids, fatty acids, minerals and vitamins [3,4]. However, the sustainable development of shrimp farming is hindered by the soaring cost of fishmeal and the scarcity of wild fish resources [5,6], driving extensive research on alternative protein sources (plant and animal proteins) [7]. Nevertheless, plant proteins (e.g., soybean meal, peanut meal) suffer from high fiber, low protein, essential amino acid (EAA) deficiency and anti-nutritional factors, which impair feed palatability, nutrient digestibility and shrimp gastrointestinal health [[8], [9]]. animal proteins (e.g., meat and bone meal, poultry by-product meal) may cause EAA imbalance and hepatic burden, posing adverse effects on liver health [[10], [11]]. As alternative proteins are inferior to fishmeal in EAA balance, functional amino acid supplementation has become a common strategy in aquatic feed formulation [12], in line with the broader principle of nutritional profiling that emphasizes balanced nutrient matching for optimal aquatic animal performance [[13], [14]].

Lysine, methionine and arginine are commonly the most limiting EAA in commercial shrimp feed [15]. In most alternative protein sources, lysine is the first-limiting amino acid [16], lysine is critical for peptides, protein and non-peptide molecular synthesis, as well as regulating body endocrinology, intermediate metabolism, reproduction and immune response [17]. Several studies have demonstrated that lysine regulates lipid metabolism, improves health, and promotes growth and immunity in aquaculture species [[18], [19], [20], [21]]. Methionine plays an important role in numerous metabolic processes, which could convert to cystine in methylation reactions for most vital protein and choline synthesis, it is also considered one of the first limiting or indispensable amino acids in practical diets [22]. Previous researches have proven that methionine can maintain intestinal health, as well as improve the protein utilization, growth, immunity and hemoglobin concentration in aquatic animals [[23], [24], [25], [26]]. Arginine is indispensable for normal growth of shrimp and functions as a phosphagen in crustaceans, and it is considered the most-limiting EAA in penaeid shrimp diets [27]. Arginine deficiency reduces growth and protein retention as shown in fish such as black sea bream [28].

A nutrient-based feed formulation allows rapid dietary adjustment via amino acid supplementation, thereby achieving the targeted EAA profiles. In Atlantic salmon (*Salmo salar L.*), supplementation of certain crystalline amino acids has been shown to elevate feed intake and significantly improve growth performance [29]. Meanwhile, in low-fishmeal diets (3%), supplementation with an amino acid mixture similar to krill extract has been shown to regulate feed intake [30]. For Yellow River Carp, a complex amino acid attractant consisting of 0.4% alanine, 0.5% arginine, and 0.7% glycine shows better suitability when applied in fishmeal‑free diets [31]. Although the individual nutritional requirements of lysine [32], methionine [33] and arginine [24] for *L. vannamei* have been reported, a critical knowledge gap still exists. The effects and regulatory mechanisms of combined supplementation of these three key EAAs in low-fishmeal diets remain unclear. This limits the rational formulation of low-fishmeal diets for *L. vannamei*.

In this study, we developed a specific amino acid mixture (NutriMix) composed of lysine, methionine and arginine—the three most limiting EAAs for *L. vannamei*. Our previous study [34] has systematically verified that this lysine-methionine-arginine mixture can improve growth performance, non-specific immunity and intestinal health in *Apostichopus japonicas*, providing a theoretical basis for its application in aquatic animal feed. Based on the above background, the present study aims to: (1) evaluate the effects of different levels of NutriMix supplementation on the growth performance, non-specific immunity and intestinal health of *L. vannamei* fed with basal and low-fishmeal diets; (2) determine the optimal supplementation level of NutriMix in basal and low-fishmeal diets; (3) reveal the underlying mechanisms by analyzing immune-related gene expression, antioxidant enzyme activities, and gut microbiota composition. We hypothesized that NutriMix could alleviate the negative impacts of low-fishmeal diets by balancing EAA supply, and improve the growth, antioxidant capacity, immunity, and intestinal health of *L. vannamei* in a dose-dependent manner, with an optimal level for practical aquaculture. This study provides a scientific basis for the rational use of EAA mixtures in low-fishmeal diets for *L. vannamei* and supports the sustainable development of shrimp culture.

## Material and methods

### Experimental diet composition

A type of amino acid mixture called NutriMix was prepared by Yumin Tech (Shandong, China). The mixture comprised three essential amino acids in the following proportions: arginine (42%), lysine (39%), and methionine (19%), a profile that approximates the essential amino acid requirements of black tiger shrimp (*Penaeus monodon*) [35]. Two diet series were designed to evaluate the effects of NutriMix under normal‑fishmeal and low‑fishmeal diet conditions. Six experimental diets were prepared with graded levels of the amino acid mixture. The formulations and proximate compositions are presented in Table 1. The positive control diet (CT) contained 200 g kg⁻¹ fishmeal and 220 g kg⁻¹ soybean meal, without amino acid supplementation. Diets AL, AM, and AH shared the same fishmeal and soybean meal levels as CT, and were supplemented with 0.5, 1, and 2 g kg⁻¹ NutriMix, respectively. Diets FM and FH contained a reduced fishmeal level (120 g kg⁻¹) and increased soybean meal (300 g kg⁻¹), supplemented with 1 and 2 g kg⁻¹ NutriMix, respectively. The 0.5 g kg⁻¹ inclusion level was omitted in the low‑fishmeal series due to its ineffectiveness observed in preliminary trials.

**Table 1:** Ingredient composition and proximate analysis of the experimental diets (g kg^−1^).

Ingredients (g kg^−1^ in dry weight)	Test diets					
	CT	AL	AM	AH	FM	FH
Fish meal	200	200	200	200	120	120
Corn Protein Powder	120	120	120	120	120	120
Wheat Gluten Meal	50	50	50	50	50	50
Soybean Meal	220	220	220	220	300	300
Wheat Flour	240	240	240	240	240	240
Fish Oil	14	14	14	14	14	14
Soybean Oil	20	20	20	20	20	20
Soy Lecithin	20	20	20	20	20	20
Cholesterol	5	5	5	5	5	5
Vitamin premix a	10	10	10	10	10	10
Mineral premix b	10	10	10	10	10	10
Bentonite	65	64.5	64	63	64	63
Yeast Extract	10	10	10	10	10	10
BHT	0.5	0.5	0.5	0.5	0.5	0.5
Vitamin C	3	3	3	3	3	3
Choline Chloride	2.5	2.5	2.5	2.5	2.5	2.5
Calcium phosphate	10	10	10	10	10	10
Amino acid mixture	0	0.5	1	2	1	2
Total	1000	1000	1000	1000	1000	1000
Nutrient levels (g kg^−1^ of dry matter)						
Crude protein	399	398.1	399.6	397.8	375	378.1
Crude lipid	74.9	75.6	75.7	75.9	66.2	62.3
Moisture	71.8	70.5	72.4	71.4	77.2	75.6
Crude ash	114.4	113.3	114	114	104.9	102.1

a Vitamin mixture (mg g^−1^ premix): vitamin A, 1400 IU; vitamin D_3_, 260 IU; vitamin E, 10; vitamin K_3_, 2; vitamin B_1_, 1; vitamin B_2_, 1.5; vitamin B_6_, 1.5; vitamin B_12_, 0.5; vitamin C, 40; nicotinic acid, 6; ca pantothenate, 3; folic acid, 0.2; inositol, 8; carrier glucose, H_2_O ≤10%.

b Mineral mixture (mg g^−1^ premix): NaCl, 116.7; MgSO_4_·7H_2_O, 426.3; NaH_2_PO_4_·2H_2_O, 256.8; KH_2_PO_4_, 680.2; Ca(H_2_PO_4_)_2_·2H_2_O, 376.7; Fe Citrate, 84.56; Ca Lactate, 907.10; Al(OH)_3_, 0.49; ZnSO_4_·7H_2_O, 12.8; CuSO_4_, 3.2; MnSO_4_·7H_2_O, 3.47; Ca(IO_3_)_2_, 0.23; CoSO_4_·7H_2_O, 3.24.

### Shrimp and experimental conditions

*L. vannamei* were procured with Shandong Yantai Marine Aquaculture Farm and reared in an indoor recirculating aquaculture tank system at the Muping Coastal Environment Research Station, Chinese Academy of Sciences (Yantai, China). Before the formal experiment began, shrimp were fasted for 24 h, and 540 healthy shrimp larvae of uniform quality were randomly selected and divided into six groups with three replicates per group and 30 shrimp per replicate. Based on previous studies [36], shrimp were fed 4 times a day (6:00, 11:00, 16:00, 21:00) for 42 days. During the feeding period, continuous aeration was maintained, with water temperature at 27 ± 2°C, salinity between 28-30‰, pH between 8.0 - 8.2, and the dissolved oxygen content remained above 6.5 mg L^−1^.

### Sample collection

At the end of the feeding trial, all *L. vannamei* from each tank were counted and weighed to calculate WGR and SGR after 24 h of starvation. Six shrimp of uniform size were randomly sampled from each tank and pooled into two technical replicates, and three replicate tanks were used as biological replicates (n = 3) for statistical analysis. The *L. vannamei* were dissected to collect hemolymph, hepatopancreas, gill, and intestinal tissues. The hemolymph was mixed with an equal volume of anticoagulant (0.4 M NaCl, 0.019 M KCl, 0.02 M EGTA, 0.068 M Tris-HCl, pH 7.6), and the hemocyte count per milliliter was determined. The hemocytes was utilized for assays measuring ROS, phagocytosis and apoptosis rate. The hepatopancreas and gill tissues were mixed with 1 ml Takara RNAiso Plus for qRT-PCR analysis. The hepatopancreas and intestines of each group were collected for determination of enzyme activity. Additionally, the intestines were collected under sterile conditions for intestinal microbiota sequencing analysis.

### Non-specific Immune Parameters Analysis

#### Flow cytometry

The reactive oxygen species assay kit (Beyotime; catalog no. S0033) and the annexin V-FITC binding apoptosis assay kit (Beyotime; catalog no. C1062L) were used to evaluate the activities of reactive oxygen species (ROS) and apoptosis according to the manufacturer’s protocols. Briefly, the intracellular ROS level was determined using the 2’7’-dichlorofluorescein diacetate (DCFH-DA) fluorescent probe. Hemocytes were incubated with 1 mL of 10 μM DCFH-DA at 37°C in the dark for 20 min, and then washed with PBS to remove unincorporated probe. Fluorescence intensity was measured at 488 nm excitation and 525 nm emission. For the detection of cell apoptosis, take 50,000 cells, centrifuge at 1000 g for 5 min, discard the supernatant, and resuspend gently in 195 μl Annexin V-FITC binding buffer. Add 5 μl Annexin V-FITC and 10 μl propidium iodide (PI) staining solution sequentially, mix gently each time, and incubate at 20-25°C in the dark for 10-20 min (resuspend cells 2-3 times during incubation). Detect immediately by flow cytometry. Phagocytosis was quantified using fluorescent microspheres (Sigma; catalog no.12162002). In short, hemocytes were incubated with 2 μm, 0.5% microspheres at a 1:1 ratio for 30 minutes at 37°C. After incubation, cells were washed to remove non-phagocytosed microspheres. The phagocyte activity was then assessed by flow cytometry, measuring the percentage of hemocytes containing ingested microspheres. Prior to loading, samples were filtered to remove aggregates. During data analysis, cell debris and irrelevant cell lineages were excluded by gating.

#### Enzyme Activity

The hepatopancreas and intestines (0.1g) were homogenized in 0.9% normal saline (0.9g) using an IKA homogenizer set to 6000 rpm and centrifuged at 5000 g for 5 min at 4°C to obtain the supernatants. Enzyme activities were determined using commercial assay kits (Jiancheng Bioengineering Institute, Nanjing, China) according to the manufacturer’s protocols. The measured parameters included: antioxidant markers - malondialdehyde (MDA), peroxidase (POD), total antioxidant capacity (T-AOC), glutathione peroxidase (GSH-Px), and superoxide dismutase (SOD); metabolic enzymes - alkaline phosphatase (AKP) and acid phosphatase (ACP); and digestive enzyme - lipase (LPS). Protein concentrations were determined using a BCA protein assay kit (Beyotime; catalog no. P0009).

### RNA extraction and quantitative real-time PCR analysis (qRT-PCR)

The qRT-PCR analysis was conducted following our previously established pipeline [37]. Briefly, total RNA of hepatopancreas and gill tissues were extracted with Trizol reagent (Invitrogen, USA). In brief, samples were lysed in TRIzol, mixed with chloroform, and centrifuged to separate the aqueous phase. RNA was then precipitated with isopropanol, washed with 75% ethanol, and finally dissolved in RNase-free water. The concentration and quality of RNA were examined with NanoDrop 2000 spectrophotometer (Thermo Fisher Scientific, USA) and 1% agarose gel electrophoresis. Only RNA sample with OD260/280 ratio between 1.8 and 2.0, and with OD260/230 ratio ≥2.0 were selected to synthesize cDNA using reverse transcription. The obtained cDNA was used as the template for real-time fluorescent quantitative PCR. Amplification efficiencies of the target genes and the reference gene were validated before formal detection. The thermal cycling conditions were set as follows: pre-denaturation at 95°C for 3 min, followed by 40 cycles of denaturation at 95°C for 10 s and extension at 60°C for 30 s. Fluorescence signals were collected during each cycle for quantitative analysis. The primer sequences of the *ef-1α, pen-3α, qm, SOD* and *GPX* were listed in Table 2. The relative expression levels of selected genes were calculated by 2^−ΔΔCT^ [38].

**Table 2:** Primers used for real-time qPCR.

Target genes	Forward primer sequence (5′-3′)	Reverse primer sequence (5′-3′)	Source
*ef-1α*	ACCAGGGACAGCCTCAGTAAG	GTATTGGAACAGTGCCCGTG	JF288785
*Pen-3α*	CACCCTTCGTGAGACCTTTG	AATATCCCTTTCCCACGTGAC	Y14926
*qm*	TCGTGTGCTGGTGCTGATAGAT	GCCTCAATGACCTGCTCCTTGT	jx880087
*SOD*	AGCCAATGACGTAAGCG	ACCATCACAAGAAACCC	HM371157
*GPX*	GGCACCAGGAGAACACTAC	CGACTTTGCCGAACATAAC	XM_027368745

### Analysis of Intestinal Microbiota

Total DNA of gut microbes of shrimps was extracted using a FastDNA SPIN Kit (MP Biomedical). The V4 region of the 16S rRNA gene was amplified using the barcoded fusion primers 338F (5′- ACTCCTACGGGAGGCAGCAG-3′) and 806R (5′- GGACTACHVGGGTWTCTAAT-3′). High-throughput sequencing (Illumina MiSeq PE300 platform, Illumina, USA) was performed by Shanghai Majorbio Bio-Pharm Technology Co., Ltd. (Shanghai, China). Raw reads were quality-filtered with a minimum quality score of 20 and a minimum read length of 200 bp. Chimeric sequences were removed, and clean reads were clustered into operational taxonomic units (OTUs). Alpha diversity (Chao1, Shannon, Simpson, ACE) and beta diversity (PCoA, NMDS) indices were analyzed for microbial community assessment. The resulting MiSeq sequencing data were processed and analyzed using the QIIME Pipeline, following our standard operating procedures [39].

### Calculations and statistical analysis

Survival rate (SR) was calculated as the percentage of initial shrimp that survived until the end of the experiment:

SR (%) = N_i_/N_0_ × 100%

where N_0_ = initial number of shrimp per tank; N_i_ = final number of shrimp per tank.

Weight gain rate (WGR) reflects the percentage increase in body weight over the experimental period:

WGR (%) = (W_i_- W_0_)/W_0_ × 100%

where W_0_ = initial body weight (g); W_i_ = final body weight (g).

Specific growth rate (SGR) was used to express the daily percentage growth rate, assuming exponential growth of shrimp during the experimental period. It was calculated using the natural logarithm of body weights:

SGR (%/d) = (LnW_i_-LnW_0_) × 100%/t

where t is the experimental duration in days, and Ln denotes the natural logarithm. The logarithmic transformation linearizes the exponential growth curve and provides a more stable estimate of growth rate that is less influenced by initial size differences.

The experiment was a completely randomized design with three replicate tanks per treatment. Each tank was considered an experimental unit, and the values of all individuals within a tank were averaged to obtain a single tank mean. Due to the incomplete factorial design (the low-fishmeal diet was not combined with the 0‰ and 0.5‰ NutriMix levels), a standard two-way ANOVA was not feasible. Therefore, the six treatments were considered as independent groups and analyzed by One-Way analysis of variance (ANOVA) with treatment as the fixed factor. When a significant overall effect was detected (*P*<0.05), Duncan's multiple range test was used for post‑hoc multiple comparisons to identify differences among individual treatment means. All results are presented as mean ± standard deviation (S.D.) based on tank replicates (*n*=3 per treatment). The significance level was set at *P* < 0.05.

## Results

### Growth performance

Among all treatments, the AH group (2‰ NutriMix) exhibited the best growth-promoting effect, as evidenced by the highest WGR and SGR (*P* < 0.05) Table 3. No significant differences were observed between the FM, FH, and CT groups, these results suggest that supplementation with 1‰–2‰ NutriMix in the low-fishmeal diet restored shrimp growth to normal levels.

**Table 3:** Growth performance of *L. vannamei* fed with six different levels diet. Different letters indicate significant differences (*P* < 0.05), and the same letter indicates no significant difference. SGR, specific growth rate; WGR, weight gain rate; SR, survival rate.

Index	Group					
	CT	AL	AM	AH	FM	FH
Initial weight (g)	2.4±0.28	2.47±0.32	2.44±0.26	2.43±0.34	2.45±0.3	2.5±0.29
Final weight (g)	13.67±1.41^a^	14.49±1.27^a^	15.21±1.27^b^	15.9±1.61^b^	13.98±0.82^a^	13.93±1.12^a^
SGR (%/day)	0.041±0.0042^a^	0.042±0.004^ab^	0.044±0.0026^ab^	0.045±0.0032^b^	0.042±0.0036^a^	0.041±0.0037^a^
WGR (%)	4.79±1.04^a^	4.98±1.03^ab^	5.28±0.72^ab^	5.61±0.89^b^	4.8±0.91^a^	4.66±0.9^a^

### Hemocytes immune parameters

**Fig. 1:**
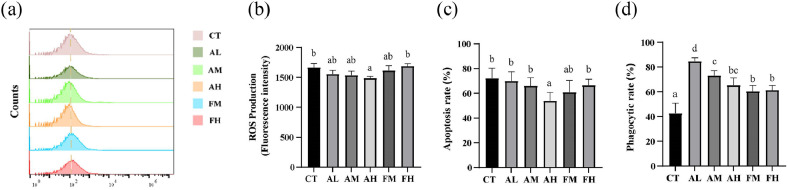
Hematocytes immune parameters of *L. vannamei* fed with six diets. (a, b) ROS production. (c) Apoptosis. (d) Phagocytosis. Different letters indicate significant differences (*P* < 0.05), and the same letter indicates no significant difference.

The ROS production in the AL group, AM group and AH group was significantly lower than that in the CT group, with the AH group also showing a significantly lower ROS level than the FH group (*P* < 0.05, Fig. 1a and 1b). The apoptosis rate in the AH group was significantly lower than that in the other groups (*P* < 0.05, Fig. 1c), whereas the FM and FH groups showed no significant differences compared with the CT group. The phagocytic rate was significantly increased in all NutriMix-supplemented groups relative to the CT group, with the AL group exhibiting the highest value among the supplemented groups (*P* < 0.05, Fig. 1d). Collectively, these results indicate that NutriMix supplementation enhanced hemocyte immune function, effectively compensating for the immune impairment induced by low-fishmeal diets to normal levels, although no dose-dependent effect was observed.

### Antioxidant defense

**Fig. 2:**
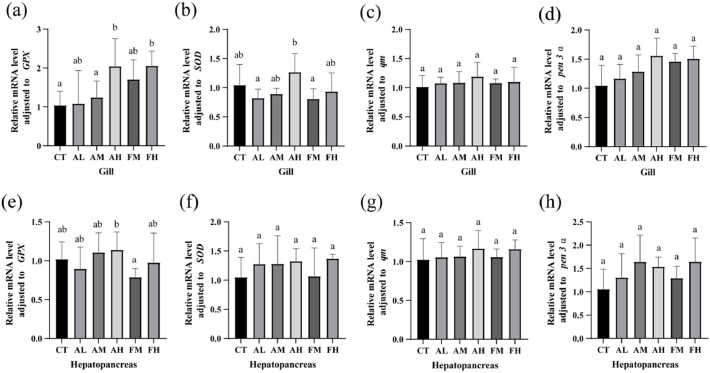
Antioxidant and immune-related gene expression of gills (a-d) and hepatopancreas (e-h) fed with six diets. Different letters indicate significant differences (*P* < 0.05), and the same letter indicates no significant difference.

**Fig. 3:**
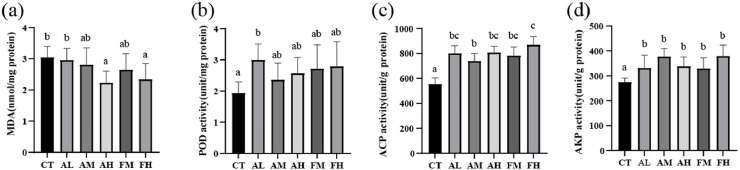
Enzyme activity in hepatopancreas of *L. vannamei* fed with six diets (a-d). Different letters indicate significant differences (*P* < 0.05), and the same letter indicates no significant difference.

In gills, the expression level of *GPX* mRNA in the AH and FH groups was significantly higher than that in the CT group, and the AH group also showed significantly higher expression than the AM group (*P*<0.05, Fig. 2a). The expression level of *SOD* mRNA in the AH group was significantly higher than that in the AL group (*P*<0.05, Fig. 2b). In hepatopancreas, no significant differences were observed in the mRNA expression of *GPX* and *SOD* among all groups (*P*<0.05, Fig. 2e, 2f). Regarding lipid peroxidation, the MDA content in the AH and FH groups was significantly lower than that in the CT group (*P*<0.05, Fig. 3a), meanwhile, AH group was significantly lower than that in the AL group. The POD activity in the AL group was significantly higher than that in the CT group (*P*<0.05, Fig. 3b). These results indicate that 2‰ NutriMix enhanced the antioxidant capacity of the gills and reduced lipid peroxidation in the hepatopancreas.

### Immune and metabolic function

In gills, no significant differences were observed in the mRNA expression of *qm* or *pen-3α* among the six groups (*P*>0.05, Fig. 2c and 2d). Similarly, in the hepatopancreas, no significant differences were found in the mRNA expression of *qm* or *pen-3α* among all groups (*P*>0.05, Fig. 2g and 2h). Regarding enzyme activities related to immune and metabolic function, the ACP activity in all NutriMix-supplemented groups was significantly higher than that in the CT group (*P*<0.05, Fig. 3c). Additionally, the AKP activity in all NutriMix-supplemented groups was significantly higher than that in the CT group (*P*<0.05, Fig. 3d), indicating that NutriMix consistently enhances the immune response of shrimp under different dietary conditions.

### Intestines enzyme activity

**Fig. 4:**
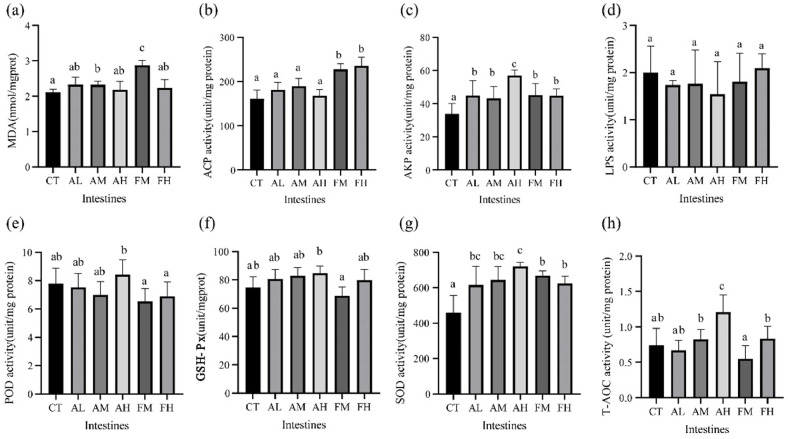
Enzyme activity in intestines of *L. vannamei* fed with six diets (a-h). Different letters indicate significant differences (*P* < 0.05), and the same letter indicates no significant difference.

There was no significant difference in MDA content and POD activity among all groups (*P* > 0.05, Fig. 4a, 4e). The GSH-Px and T- AOC activity showed that the AH group was significantly higher than the CT group, while the FM group was significantly lower than the other amino acid mixture groups (*P* < 0.05, Fig. 4f, 4h). These results indicate that, in the low-fishmeal diet, the antioxidant effect of 1‰ NutriMix was not evident. In SOD and AKP activity, all amino acid mixture-supplemented groups were significantly higher than that in the CT group (*P* < 0.05, Fig. 4c, 4g). In ACP activity, both FM and FH groups exhibited significantly higher levels than the CT, AL, AM and AH groups (*P* < 0.05, Fig. 4b), likely resulting from the stimulation of the low‑fishmeal diet. These results indicate that, in the normal diet, NutriMix significantly enhances the intestinal antioxidant and immune capacity of shrimp. In LPS activity, the AL group was significantly lower than the CT, AM, AH and FH groups (*P* < 0.05, Fig. 4d), indicates a nonlinear dose–response.

### Intestinal microbiota analysis

#### Alpha diversity analysis

**Fig. 5:**
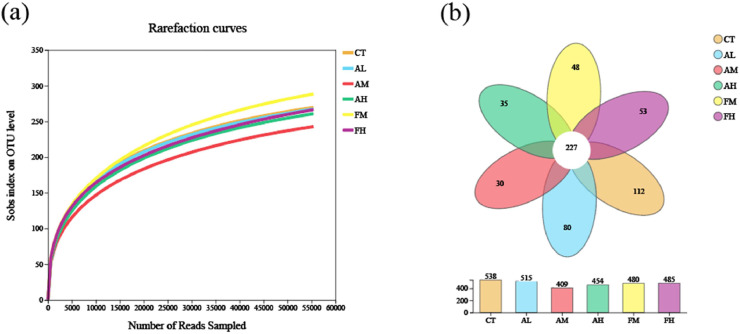
(a) Rarefaction curves of gut samples from *L. vannamei*. (b) Venn diagram of *L. vannamei* fed with six diets.

Quality control and optimization of raw data from the 30 intestinal content samples with an average length of 417 bp. At the OTU level under the Sobs index, the rarefaction curves approximately tended to a plateau above 54,579 reads, indicating that the depth of the sequencing data was sufficient to reflect the diversity of the samples (Fig. 5a). The Venn diagram showed that the six groups shared 227 OTUs. There were 112, 80, 30, 35, 48 and 53 unique OTUs in the CT, AL, AM, AH, FM and FH group, respectively (Fig. 5b).

The bacterial community alpha diversity indices are shown in Table 4. The OTUs, Chao1 and ACE indices in the FM group were significantly higher than those of the other groups (*P* < 0.05), indicating that the FM group exhibited a significant increase in gut microbial richness. Pearson correlation analysis (Fig. 9) showed that the ACE, Chao1, sobs, and OTUs indices were significantly positively correlated with the abundance of *Actibacter* (*P* < 0.05). The Simpson index in the AL group was significantly higher than that in the other groups (*P* < 0.05), and exhibited significant positive correlations with the abundances of *Ruegeria, Tenacibaculum*, and *Motilimonas*, but was negatively correlated with the abundance of *Maritimibacter* (*P* < 0.05) (Fig. 9). The Shannon index in the FM, FH, AM and AH groups was significantly higher than that of AL and CT groups (*P* < 0.05), further indicating that these groups gained many low‑abundance bacteria.

**Table 4:** Alpha diversity indices of intestinal microbiota analysis of *L. vannamei* fed with six different levels diet. Different letters indicate significant differences (*P* < 0.05), and the same letter indicates no significant difference. SGR, specific growth rate; WGR, weight gain rate; SR, survival rate.

Index	Group					
	CT	AL	AM	AH	FM	FH
OTUs	188±12.71^a^	187.6±3.36^a^	192±8.72^a^	197.8±15.61^a^	212.2±5.81^b^	200.4±10.11^a^
Shannon	2.97±0.22^b^	2.75±0.24^a^	2.98±0.14^b^	3.03±0.09^b^	3.03±0.07^b^	3.05±0.15^b^
Simpson	0.09±0.02^a^	0.12±0.03^b^	0.09±0.02^a^	0.09±0.01^a^	0.09±0.01^a^	0.09±0.02^a^
ACE	206.38±15.8^ab^	203.08±6.4^a^	209.43±9.1^ab^	215.02±15.27^ab^	231.52±10.47^b^	219.76±10.66^ab^
Chao1	207.77±17.63^a^	208.53±8.4^a^	208.02±9.1^a^	219.62±13.99^a^	232.99±16.88^b^	223.64±17.37^a^

#### Beta diversity analysis

**Fig. 6:**
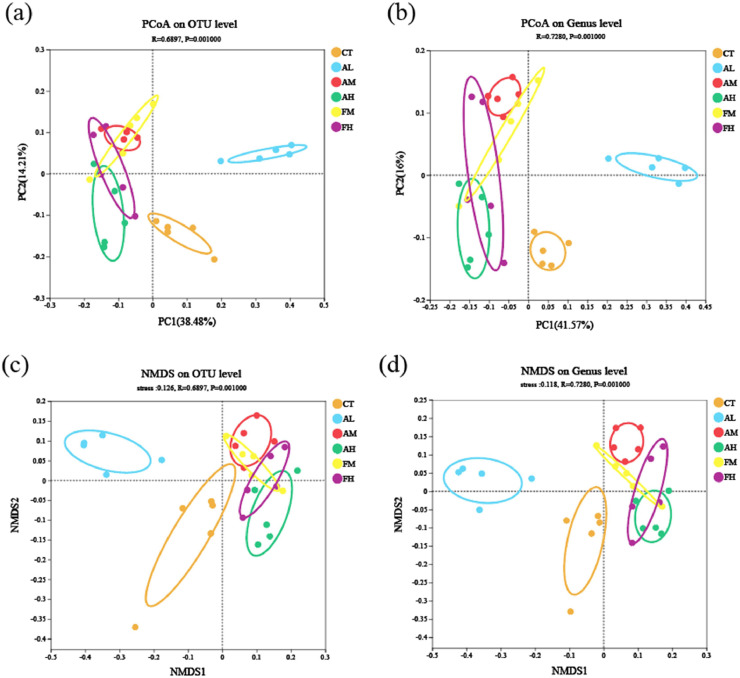
PCoA scatter plot (a-b) and NMDS scatter plot (c-d) of *L. vannamei* fed with six diets.

The beta diversity was estimated using principal coordinate analysis (PCoA) and non-metric multidimensional scaling analysis (NMDS). The PCoA analysis based on the Bray-Curtis distance disclosed significant clustering distinctions among the groups, CT clustering with AL, AM, AH, FM clustering with FH. At the OTU level, the two principal coordinates, PC1 and PC2, accounted for 38.48% and 14.21% of the variance, respectively, while at the genus level, the two principal coordinates, PC1 and PC2, accounted for 41.57% and 16% of the variance, respectively (Fig. 6a and 6b). The consistency of the result was confirmed by NMDS, which revealed the CT and AL groups showed significant differences in intestinal microbial community structures compared to the AM, AH, FM and FH groups (Fig. 6c and 6d).

#### Bacterial community analysis

**Fig. 7:**
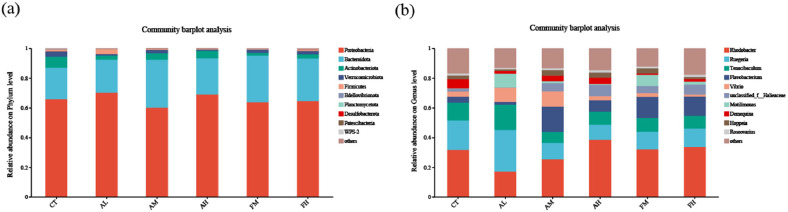
Community bar chart of *L. vannamei* fed with six diets. (a) Phylum-level community composition bar chart. (b) Genus-level community composition bar chart.

At the phylum level, Proteobacteria and Bacteroidota were the predominant bacterial phyla in the intestinal microbiota of *L. vannamei* (Fig. 7a). The relative abundance of Bacteroidota in all NutriMix-supplemented groups increased compared with the control, among them, the AM group showing the highest increase (32.36%). Bacteroidota are known polysaccharide degraders that facilitate nutrient absorption. Conversely, Actinobacteriota decreased in all supplemented groups, most markedly in the FM group (0.19%). Actinobacteriota are involved in vitamin synthesis, but their low basal abundance limits the biological impact of this reduction.

At the genus level, the intestinal microbiota of *L. vannamei* was composed of *Rhodobacter, Ruegeria, Tenacibaculum* and other dominant bacteria (Fig. 7b). *Demequina* (6.03%) was the dominant genus in the CT group. *Motilimonas* (9.23%) was the dominant genus in the AL group. *Flavobacterium* (16.84%) and *Vibrio* (10.32%) were the dominant genus in the AM group. *Vibrio* includes opportunistic pathogens. *unclassified_f_Halieaceae* (7.43%) was the dominant genus in the AH group. In the low-fishmeal groups (FM and FH), *Flavobacterium* was the dominant genus (14.17% and 13.06%, respectively), with no *Vibrio* enrichment.

Pearson correlation showed that the final weight, SGR and WGR were positively correlated with the abundance of *Lysobacter, Xanthomarina, norank_f_Rhodobacteraceae, norank_f_unclassified_o_Gammaproteobacteria_Incertae_Sedis, Lysobacter* and *Xanthomarina* are known to produce extracellular enzymes or antimicrobial compounds. The negatively correlated with the abundance of *Paracoccus, Sungkyunkwania, Haloferula*, and *Ruegeria* (*P* < 0.05, Fig. 9).

#### Microbial communities with statistically significant differences

**Fig. 8:**
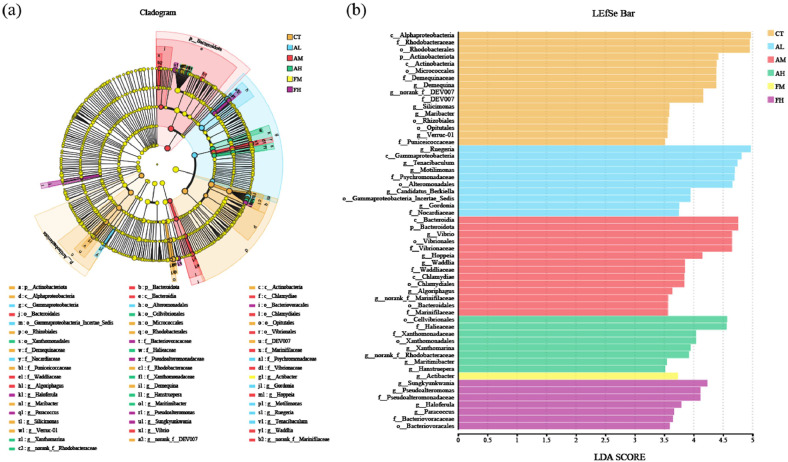
The LEfSe multi-level phylogenetic tree (a) and LDA discrimination bar chart (b) of *L. vannamei* fed with six diets.

There were sixteen, eleven, sixteen, ten, three, and seven significantly differentially abundant taxa (phylum to genus) in the CT, AL, AM, AH, FM and FH groups, respectively, identified by LEfSe analysis (LDA score > 3.5, Fig. 8a and 8b). The CT group showed significant enrichment of *Silicimonas* (phylum to genus), *Demequina* (phylum to genus) and *Verruc-01* (phylum to genus). The AL group was characterized by the dominance of two key taxa, including Gammaproteobacteria_Incertae_Sedis (phylum to order) and *Gordonia* (phylum to genus). The AM group saw significant enrichment in *Hoppeia* (phylum to genus) and *Waddlia* (phylum to genus). The main biomarkers of the AH group were Halieaceae (phylum to family) and Xanthomonadaceae (phylum to class). The FM group was predominantly associated with *Actibacter* (phylum to genus). The FH group displayed notable enrichment of *Bacteriovoracaceae* (phylum to genus) *and Haloferula* (phylum to genus).

## Discussion

The present study demonstrates that dietary supplementation with a balanced essential amino acid mixture (arginine (42%), lysine (39%), and methionine (19%)) at 2‰ (AH) significantly improves growth performance, immune function, antioxidant capacity, and intestinal health of *Litopenaeus vannamei*, even under low-fishmeal conditions. This is a key novel finding: the EAA mixture effectively compensates for fishmeal reduction, and the effect is more pronounced in normal diets than in low-fishmeal diets, where other limiting factors may still constrain performance.

The mechanism can be summarized as follows: the EAA mixture enhances systemic immunity, reinforces antioxidant defense, and modulates gut microbiota, ultimately promoting growth. First, the mixture activated a coordinated immune response. In hemocytes, a significant elevation of phagocytic rate, together with reduced apoptosis and ROS production, indicate that the mixture primes cellular immunity without causing oxidative damage [40,41]. Additionally, increased ACP and AKP activities in both hepatopancreas and intestine suggest improved immune-metabolic capacity [42].

Second, the mixture strengthened antioxidant defenses. In gills, *GPX* expression increased. GPX uses GSH to reduce H_2_O_2_ and ROOH into water and alcohols, and forms crucial defense systems against oxidative stress by maintaining redox balance [43,44]. The significant reduction of MDA in the AH and FH groups confirms effective alleviation of oxidative damage. The increase in POD activity further supports enhanced ROS scavenging. Notably, the AH group (2‰ in normal diet) showed the strongest antioxidant response, while the FH group (2‰ in low-fishmeal diet) also exhibited benefits but to a lesser extent, indicating that the response is dose-dependent and influenced by dietary background.

Third, the mixture improved intestinal health. In the intestine, it elevated T-AOC, SOD, and GSH-Px activities. It also increased ACP and AKP activities, indicating dual roles in antioxidant defense and nutrient digestion [45]. However, the activities of T‑AOC, SOD, and GSH‑Px in the FM group were significantly lower than those in the AH group, but showed no significant difference compared with the control group. The reduction in fishmeal content (which naturally provides antioxidants such as taurine and nucleotides) combined with an insufficient dosage of exogenous amino acids may have been inadequate to fully activate the antioxidant defense system [46]. Intestinal lipase (LPS) activity in the AL group was lower than the other groups, indicates a nonlinear dose–response, and no compensatory effect was observed with 1‰ or 2‰ NutriMix in the low‑fishmeal diet. This pattern could reflect a physiological trade-off, with energy being preferentially allocated to immune function—consistent with the highest phagocytic rate observed in the AL group—rather than to lipid digestion [47]. Further investigations are required to clarify the mechanisms underlying the dose-dependent effects of amino acid supplementation on digestive enzyme activity.

**Fig. 9:**
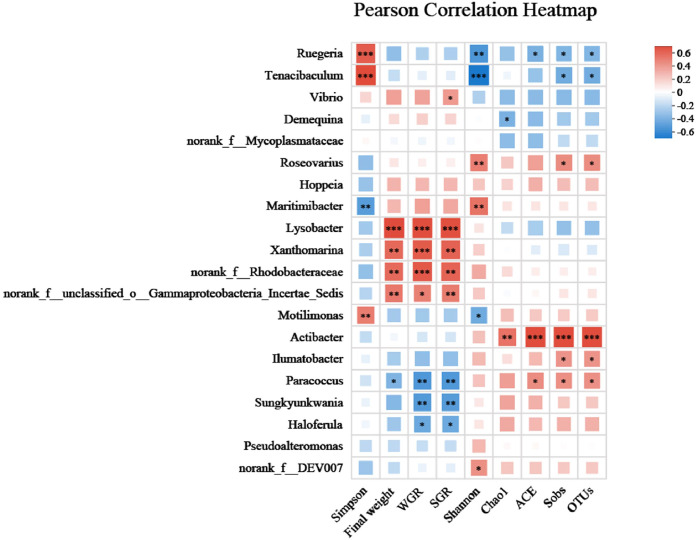
Multi-shape correlation heatmap of *L. vannamei* fed with six diets.

Fourth, the mixture modulated gut microbiota in a beneficial direction. The relative abundance of Bacteroidota increased, particularly in the AM group. Bacteroidota are known degraders of complex polysaccharides; their increase likely enhances dietary fiber digestion and short-chain fatty acid production, contributing to energy harvest. Conversely, the proportion of *Vibrio* decreased with increasing EAA dose, especially in the AH, and FH groups. This pathogen inhibition may be mediated by the enrichment of *Bacteriovoracaceae* (predatory bacteria) in the FH group, which can lyse Gram-negative bacteria. Pearson correlation analysis (Fig. 9) directly linked growth parameters to specific bacteria: final weight, SGR, and WGR were positively correlated with *Lysobacter* and *Xanthomarina. Lysobacter* produces extracellular enzymes and antimicrobial compounds [48], and *Xanthomarina* helps remove nitrogenous waste [49]. Negative correlations were observed with *Paracoccus* and *Ruegeria*, genera without known beneficial functions in shrimp. Meanwhile, the AL group showed the lowest richness but the highest Simpson index, indicating uneven distribution of the microbial community, with a limited number of taxa dominating this group. Beta diversity analysis revealed distinct clustering patterns among the AM, AH, FM, FH, AL, and CT groups, and NMDS analysis further corroborated the modulatory effects of the EAA mixture on gut microbiota structure.

A consistent finding is that the AH group (2‰ in normal diet) often outperformed the FM and FH groups (2‰ in low-fishmeal diet) in growth, immune, and antioxidant parameters. This indicates that the effectiveness of EAA supplementation is strongly influenced by basal fishmeal level. In low-fishmeal diets, other limiting factors (e.g., taurine, nucleotides, or trace elements) may still constrain performance, even when EAA balance is restored. This explains why the FH group did not achieve the same growth as the AH group. Moreover, the lack of a dose-response in low-fishmeal diets (FM vs. FH) suggests that once a certain EAA threshold is reached, additional amino acids may not further improve growth and could even impose a metabolic burden [50].

Based on these results, we recommend a dietary supplementation level of 2‰ of this EAA mixture for *L. vannamei*. This dose is effective even in low-fishmeal diets, making it a valuable tool for formulating sustainable, low-fishmeal feeds that reduce reliance on marine ingredients.

## Conclusion

In conclusion, dietary supplementation with a balanced EAA mixture at 2‰ significantly enhances growth, systemic immunity, antioxidant capacity, and intestinal health of *L. vannamei*, even under low-fishmeal conditions. The mixture improves gut microbiota by increasing beneficial polysaccharide-degrading bacteria (Bacteroidota) and suppressing potential pathogens (*Vibrio*), likely through competitive exclusion and predation by *Bacteriovoracaceae*. Growth-promoting effects are mediated in part by bacteria positively correlated with growth (*Lysobacter, Xanthomarina*). These findings provide a mechanistic basis for using EAA mixtures to replace a portion of fishmeal in shrimp feeds. Future research should investigate optimal EAA profiles for different life stages and rearing conditions, as well as the long-term safety and economic feasibility of 2‰ supplementation. This study contributes to the development of sustainable, low-fishmeal aquaculture feeds.

## Ethical approval

All animal experiments were strictly performed according to the Guidelines of the Chinese Council on Laboratory Animal Care (2001), which was approved by the Animal Care and Use Ethics Committee of the Yantai Institute of Coastal Zone Research (KJ-LL-008), Chinese Academy of Sciences.

## CRediT authorship contribution statement

**Chengjie Lv:** Methodology, Data curation. **Jiali Wu:** Writing – original draft, Methodology, Investigation. **Yongliang Liu:** Validation, Data curation. **Weiwei Zhang:** Validation, Methodology, Investigation. **Dinglong Yang:** Writing – review & editing, Validation, Funding acquisition, Formal analysis. **Jianmin Zhao:** Methodology, Investigation.

## Declaration of competing interest

The authors declare that they have no known competing financial interests or personal relationships that could have appeared to influence the work reported in this paper.

## Data Availability

Data will be made available on request.
